# How unclogging a sink can be lethal: case report of an accidental methyl bromide poisoning leading to a multiple organ failure

**DOI:** 10.1186/s40560-015-0079-2

**Published:** 2015-03-12

**Authors:** Sylvain Lecailtel, Céline Broucqsault-Dedrie, Clément Vanbaelinghem, Martine Nyunga, Delphine Colling, Patrick Herbecq

**Affiliations:** Intensive Care Unit, Victor Provo Hospital, Roubaix, France

**Keywords:** Methyl bromide, Poisoning, Multiple organ failure, Intensive care

## Abstract

Methyl bromide (CH3Br) is a colorless and odorless volatile gas, used as an insecticide, fire extinguisher, fumigant, and refrigerant. Although forbidden since 1987 for domestic use, it is still used in industry, for example, to fumigate agricultural fields which are for importation in the United States. Here is the case of a 74-year-old man who was accidentally exposed to methyl bromide after using an old fire extinguisher. Even though he finally survived, he developed a severe multiple organ failure and spent 2 months in intensive care unit. We present in this report all the difficulties we had to diagnose this unusual poisoning.

## Background

Methyl bromide (CH3Br) is a colorless and odorless volatile gas, used as an insecticide, fire extinguisher, fumigant, and refrigerant. However, as every halogen gas, it is toxic both to environment and to humans. As a consequence, the Montreal agreements have forbidden its use since 1987. Unfortunately in many countries, especially developing countries, this gas is still used. Thirty-eight cases of methyl bromide poisoning were reported in the literature. CH3Br poisoning is usually accidental, and related to chronic, or acute exposure. We present here a case report of acute accidental methyl bromide poisoning, responsible for a severe multiple organ failure (MOF).

## Case presentation

A 74-year-old man with an unremarkable medical history was referred to the Roubaix Hospital emergency department on November 13th 2012. The reasons for his consultation were headaches, vomiting, walking trouble, and dysarthria of sudden onset a few hours ago. His vital signs were blood pressure: 156/57 mmHg, heart rate: 103/min, and body temperature: 95°F (35°C). The first examination of the patient revealed cerebellar signs such as ataxia, apraxia, and dysarthria. The initial level of consciousness was normal (Glasgow Coma Score (CGS) 15). The head CT scan was normal, without ischemic or hemorrhagic stroke. Because of meningeal signs, a lumbar puncture was performed and found only 2 cells per mm^3^. The neurological investigations were completed by a brain magnetic resonance imaging (MRI), confirming the absence of ischemic or hemorrhagic stroke. A brainstem stroke was also discarded, and the basilar artery was permeable.

Few hours later, the patient deteriorated and presented coma (GCS 7), requiring intubation and mechanical ventilation. He was then transferred to our intensive care unit. The first lab results revealed a nonspecific inflammatory syndrome (white blood cells: 25,000/μL, C-RP: 96 mg/L) and renal failure (creatinine: 266 μmol/L, urea: 13.8 mmol/L). All standard toxic substances were negative (alcohol, barbiturates, benzodiazepines, tricyclic antidepressants, and carbon monoxide). The chest X-ray revealed an alveolar infiltrate of the right lung. The renal ultrasound was normal.

Initial management included mechanical ventilation, sedation, volemic expansion, broad spectrum antibiotics to treat a possible meningitis, and intravenous acyclovir to treat a possible herpetic encephalitis.

Early evolution was marked by the persistence of shock requiring vasopressive support (norepinephrine). A few hours later, the patient presented an acute coronary syndrome, complicated by severe arrhythmias, namely ventricular fibrillation, responsible for a cardiogenic shock with a left ventricular ejection fraction (LVEF) at 30%.

Because of multiorgan failure, including cerebral, renal, pulmonary, and cardiac dysfunctions, several diagnoses were suspected. Normal levels of complement system’s proteins and plasma protein electrophoresis were not in favor of an autoimmune disease. c-ANCA antibodies, anti-glomerular basement membrane antibodies, antinuclear, and lupus anticoagulant antibodies were negative, allowing to rule out Wegener’s granulomatosis, Goodpasture’s syndrome, and lupus erythematosus. Finally, a thrombotic microangiopathy was also suspected, but ADAMST 13 activity was normal. In addition, there was no hemolytic anemia, neither thrombopenia. It should be noted that viral serologies such as HIV and B and C hepatitis were also negative.

**Figure 1:**
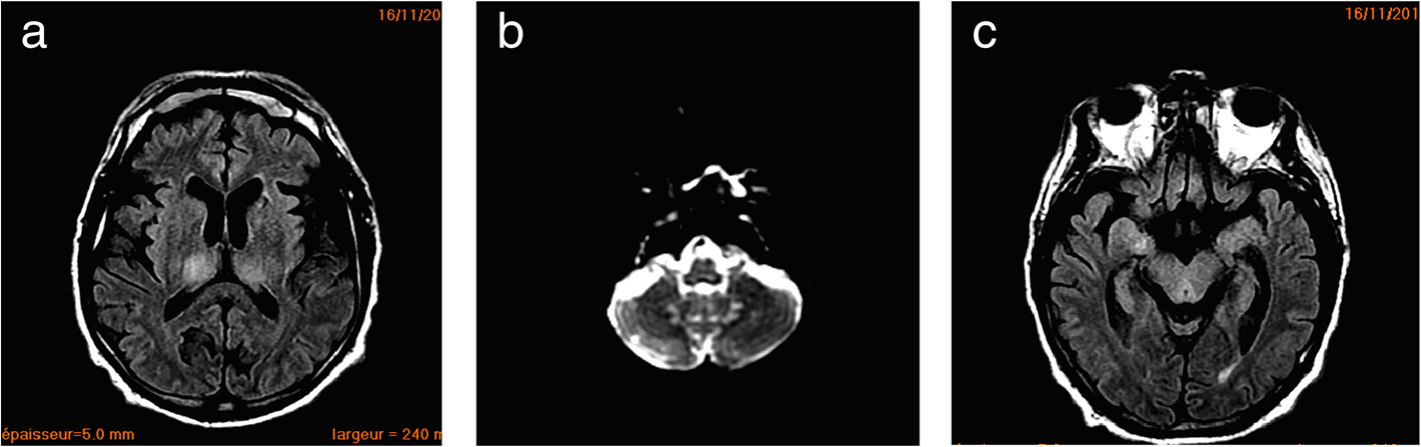
**Brain abnormalities of a patient. (a)** MRI showing hyperintense lesions of thalami. **(b)** Same patient; MRI showing hyperintense lesions of dentate nuclei. **(c)** Same patient; MRI showing lesions of the posterior white matter in the brainstem pons and in the periaqueductal gray matter of the midbrain.

After discussion with the radiologist and the neurologist, it appeared that the first MRI might have been realized too early. A second one was then performed on November 16th (day 4). It showed several abnormalities as follows: hyperintense lesions of thalami (Figure 1a), dentate nuclei (Figure 1b), posterior white matter in the brainstem pons, and periaqueductal gray matter of the midbrain (Figure 1c) on fluid-attenuation inversion recovery (FLAIR) and diffusion weighted-sequences.

**Figure 2:**
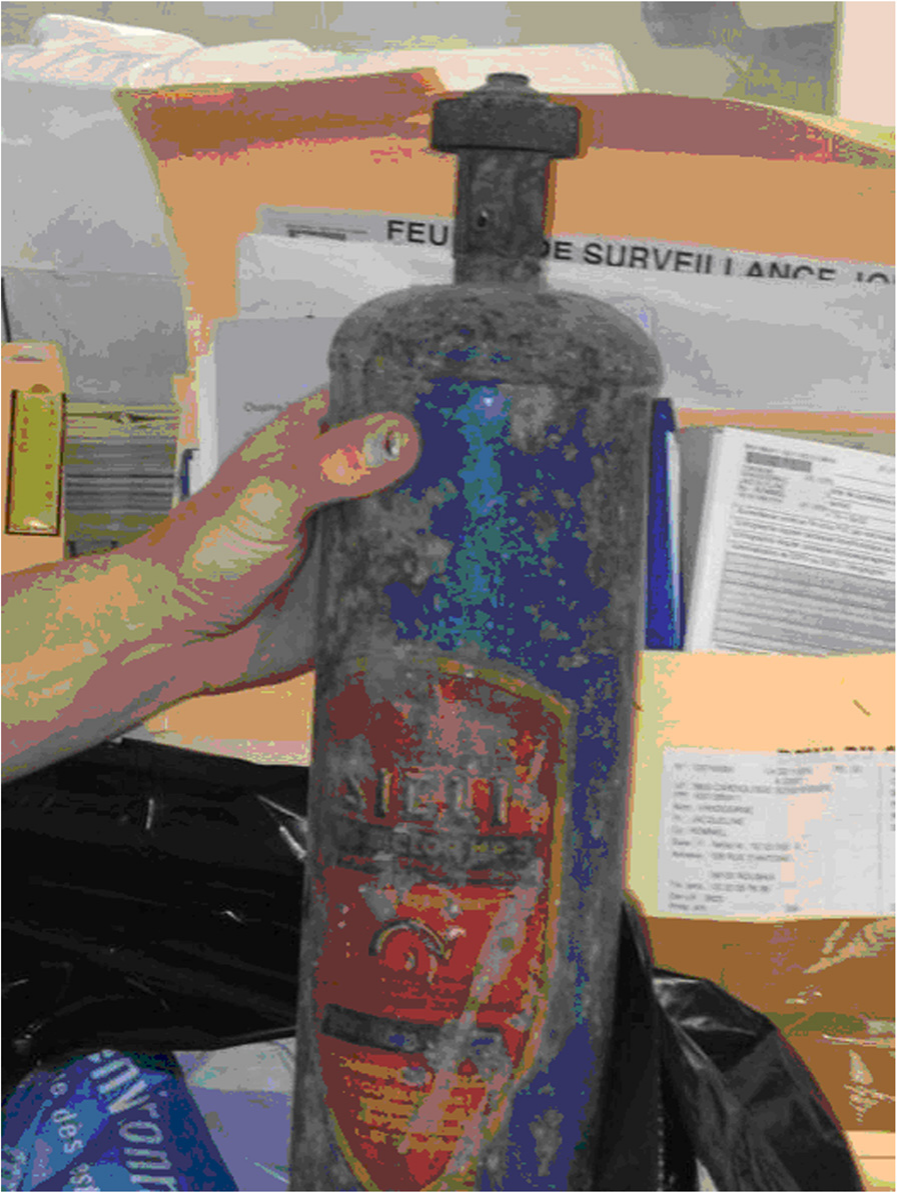
**The patient used the old fire extinguisher to unclog his sink, containing methyl bromide.**

**Figure 3:**
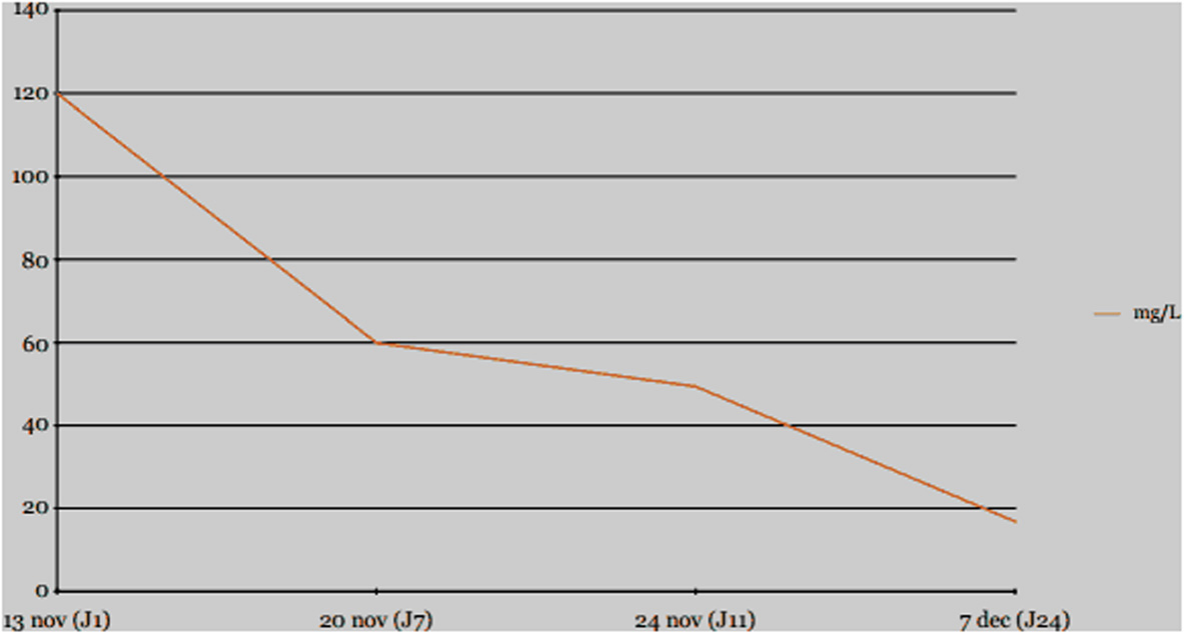
**Evolution of the methyl bromide plasma levels.**

At day 7, the son of the patient brought us the answer: an old empty extinguisher (Figure 2). The patient used it in the morning of his consultation to unclog his sink and he inhaled the content of the extinguisher, which contained methyl bromide. A blood sample was sent to specialized toxicology laboratory in Paris. The methyl bromide blood level was over 120 mg/L (normal level <0.05 mg/L). Assuming a 12-day half-life for methyl bromide [1], his serum bromide was probably much higher at his admission (7 days before the blood sample). Four weeks later, methyl bromide was still positive in the patient’s blood (Figure 3).

Several authors have studied methyl bromide’s neurotoxicity and reported similar brain MRI results. Bilateral strikingly symmetrical high signal intensity on T2-weighted and FLAIR sequences have been reported in the bilateral dentate nuclei of the cerebellum, periventricular area of the fourth ventricle, dorsal medulla (inferior olivary nuclei, restiform bodies, vestibular nuclei), dorsal pons, periaqueductal gray matter, superior and inferior colliculi, posterior putamen, subthalamic nuclei, and corpus callosum. These lesions were hypointense on T1-weighted images and devoid of mass effect. Post-gadolinium studies demonstrated no enhancement [2-6]. These lesions were thought to be related to local injury due to ischemia resulting from vasospasm, to increased axonal and interstitial water content, and also to a direct effect of the methyl bromide on Purkinje cells’ nucleic acid [7,8].

### Evolution

As regards to the cardiovascular system, the patient presented an acute coronary syndrome at day 2, responsible for a cardiogenic shock. The diagnosis was made based on the combination of an elevated troponin up to 54 μg/L and changes in the 12-lead electrocardiogram. There was indeed a *de novo* ST elevation and a Pardee wave in the inferolateral territory, with mirror images in the anterior territory. The transthoracic echocardiography revealed kinetics abnormalities in the corresponding territories and a decreased left ventricle ejection fraction at 30%. The patient also presented ventricular fibrillation, reversed after three external electric shocks. Given the fact that the patient was highly unstable, presented acute renal failure, and in the hypothesis of a septic shock, we decided not to perform an angiocoronarography immediately. The cardiogenic shock was controlled after 4 days of cardiotropic treatment by dobutamine, and the electrocardiogram was back to anterior state within 48 h, under cover of a treatment by double platelet anti-aggregation. One month later, the LVEF increased at 50%.

The angiocoronarography performed around day 40 found normal coronary arteries. The assumption of a myocardial infarction induced by the methyl bromide himself was then not confirmed. The major etiological hypothesis for this cardiac and/or coronary distress is a side effect of the severe initial shock with low cardiac output. Indeed, initial cardiac ultrasound showed localized kinetics abnormality, well correlated with electrocardiogram, which did not suggest a stress cardiomyopathy such as Takotsubo (no contrast between apex and basis of the left ventricle). A direct cardiotoxicity of the methyl bromide is quite unlikely because no similar case has been described in the literature.

As regards to renal failure, the urinary balance showed a proteinuria at 2.15 g/day. The renal ultrasound showed no obstruction of the urinary tract, two kidneys of normal size, and a good corticomedullary differentiation.

The renal function got worse to reach a real anuria with creatininemia at 575 μmol/L and ureamia at 38,2 mmol/L. Extrarenal epuration was required for 11 days. We used a continuous venovenous hemofiltration instead of intermittent dialysis because of hemodynamic unstability. A normal renal function was finally reached in 16 days, with a creatininemia at 90 μmol/L, and a normal urinary output. This renal failure seems to be of renal type. On the first blood sample from the emergency ward, creatinine level is already more increased than the urea is. The natriuresis was never locked. Moreover, the early volemic expansion and hemodynamic stabilization had no effect on renal function and diuresis. This renal failure can easily be explained by the methyl bromide himself [9].

As regards to respiratory failure, ventilator-associated pneumonia was suspected at day 4. The patient presented hyperthermia at 101.3°F (38.5°C), an important inflammatory syndrome with C-RP at 195 mg/L and a right alveolar infiltrate on the chest X-ray. Two days later, in spite of a broad-spectrum antimicrobial treatment (Tazocilline), it progressed to acute respiratory distress syndrome (ARDS) with a PaO2/FiO2 ratio at 80 and bilateral diffuse alveolar syndrome on the chest X-ray. There was no argument in favor of a cardiogenic pulmonary oedema, in spite of the concomitant ventricular dysfunction, because the left ventricular filling pressures were low. The patient required specific ARDS ventilation and curarization. Finally, ARDS quickly resolved, and the patient did not require prone positioning. The pulmonary evolution was also marked by the occurrence at day 8 of a pneumothorax under mechanical ventilation, which had to be been drained by a chest tube.

All the respiratory events were obviously not the result of direct injury of the intoxication but were induced by the neurological injury, requiring intubation and mechanical ventilation.

Delayed weaning from the ventilator was partly related to ventilator-associated pneumonia. In addition, the patient presented a polyneuropathy with tetraparesia. ICU-acquired weakness or “critical illness polyneuropathy” was confirmed by an electromyogram showing a peripherical neurogenic disturbance. It was certainly favored by 56 days of mechanical ventilation and the severity of the multiple organ failure [10]. However, this kind of peripheral neuropathy has also been frequently described in cases of methyl bromide poisoning [11-14]. On top of this, the methyl bromide intoxication resulted in central neurological effects, such as delayed awakening, and alertness disorders. Moreover, the patient experienced swallowing disorders, probably due to the methyl bromide intoxication itself [3,15]. A percutaneous tracheotomy was performed because of weaning difficulties.

Finally, the patient was decanulated after 56 days of mechanical ventilation. A brain MRI, performed 6 months later, showed that most of the lesions had improved. It remained only sequels on the left internal capsule and specifically the thalamus and the dentate nucleus. According to the literature, brain MRI lesions due to methyl bromide toxicity are frequently reversible [7,8,16]. He has been transferred to a rehabilitation department specialized in rehabilitation of neurological patients. To date, he is back home. After hours of physical and speech therapy, he still faces sequels such as dysarthria and ataxia and needs a Zimmer to walk by himself.

As far as pathophysiology is concerned, several mechanisms seem to be the cause of the methyl bromide toxicity. Most of the authors have described a cellular toxic effect. Indeed, following a methylation reaction, the methyl bromide conjugates itself with glutathione to make methylglutathione, which is highly toxic for the respiratory chain [17-20]. A second cause often described is the modulation of the inhibitory neurotransmitter γ-aminobutyric acid receptor within the cerebellar and vestibular systems [9,16,21]. A third hypothesis attributes toxicity to methyl bromide metabolites such as methanethiol and formaldehyde [17,19]. Another suggested mechanism is that methyl bromide rapidly inhibits creatine kinase activity in all regions of the brain and in other target organs. Creatine kinase is an enzyme catalyzing the conversion of adenosine triphosphate and creatine to adenosine diphosphate and phosphocreatine [22,23].

## Conclusion

Cases of methyl bromide poisoning have become rare since his prohibition in 1987 thanks to the Montreal agreements. These cases being rare, it makes it even harder to establish the proper diagnosis. The clinical presentation of the acute methyl bromide poisoning can be tricky. However, common clinical signs have been reported, such as headaches, vomitings, cerebellar signs, and seizures [24]. Our patient presented all of them, except for seizures, which are however quite frequent in this intoxication [4]. Several complications have been described such as ARDS and acute kidney injury. Our patient experienced all these complications. Some authors reported the use of extrarenal epuration [25,26], but they highlight the importance of the earliness of this treatment. In chronic intoxication, signs are quite different. Neuropathies, liver insufficiencies, kidney insufficiencies, and neuropsychiatric disorders were reported [2].

However, no antidote is available. Many treatments have been tested such as Glucagon or *N*-Acetylcysteine but are ineffective [27,28].

## Consent

Written informed consent was obtained from the patient for publication of this case report and any accompanying images. A copy of the written consent is available for review by the Editor in Chief of this journal.

## Abbreviations

ARDS: Acute respiratory distress syndrome
C-RP: C-reactive protein
CT: Computer tomography
HIV: Human immunodeficiency virus
LVEF: Left ventricular ejection fraction
MOF: Multiple organ failure
MRI: Magnetic resonance imaging
